# Reliability of non-contrast magnetic resonance angiography-derived aortic diameters in Marfan patients: comparison of inner vs. outer vessel wall measurements

**DOI:** 10.1007/s10554-020-01850-4

**Published:** 2020-04-20

**Authors:** Julius Matthias Weinrich, Maxim Avanesov, Alexander Lenz, Enver Tahir, Frank-Oliver Henes, Bjoern Philip Schoennagel, Meike Rybczinsky, Gerhard Adam, Yskert von Kodolitsch, Peter Bannas

**Affiliations:** 1 Department of Diagnostic and Interventional Radiology and Nuclear Medicine, University Medical Centre Hamburg-Eppendorf, Martinistr. 52, 20246 Hamburg, Germany; 2 Department of General and Interventional Cardiology, University Medical Centre Hamburg-Eppendorf, Hamburg, Germany

**Keywords:** Marfan syndrome, Magnetic resonance angiography, Aortic aneurysm

## Abstract

Aortic diameter measurements play a crucial role for the indication of aortic root surgery in Marfan patients. However, for magnetic resonance angiography (MRA)-derived measurements, there is no consensus on whether the aortic wall should be included or excluded in the aortic diameter. The purpose of this retrospective study was to compare the reliability of non-contrast bright blood MRA aortic inner-to-inner and outer-to-outer edge measurements in patients with Marfan syndrome. Forty Marfan patients underwent ECG-gated balanced steady-state free-precession MRA of the aorta at 1.5 T. Two readers independently performed inner and outer measurements at different aortic levels. They rated the image quality of the delineation of both inner and outer vessel wall edges on a four-point scale. MRA-derived diameters of the sinuses of Valsalva were compared with echocardiography-derived diameters. Aortic vessel wall delineation score was rated higher at all levels for inner than for outer vessel walls (p < 0.001). Inter- and intraobserver variances of aortic measurements were smaller for inner-to-inner measurements at the sinuses of Valsalva, sinotubular junction and ascending aorta (p < 0.03). There was a difference of 1.1 ± 2.3 mm for inner MRA measurements (p = 0.014) and 6.9 ± 3.1 mm for outer MRA measurements (p < 0.001) when compared to echocardiographic leading-edge measurements. Inner-to-inner vessel wall diameter measurements in non-contrast bright blood MRA provide more reliable diameters when compared to outer-to-outer vessel wall measurements of the aortic root. Therefore, we propose to rely on inner rather than outer aortic wall measurements in non-contrast-MRA when monitoring aortic diameters in patients with Marfan syndrome.

**Electronic supplementary material:**

The online version of this article (10.1007/s10554-020-01850-4) contains supplementary material, which is available to authorized users.

## Introduction

Marfan syndrome is a hereditary connective tissue disorder caused by mutations in the *FBN1* gene encoding the protein fibrilin-1 [1]. The prevalence of this autosomal-dominant inherited disease is one in 500–10,000 individuals [2, 3]. Progressive dilation of the aortic root is the most common cardiovascular complication of Marfan syndrome [1, 4]. The connective tissue disorder affects different parts of the human body, including heart, blood vessels, lungs, skin, bones, joints, and eyes [3, 5]. Aortic root aneurysms may cause aortic dissection and represent the main cause of death in Marfan syndrome [1, 4, 6, 7].

Timely diagnosis of aortic root aneurysms in Marfan patients allows for prophylactic aortic surgery to evade the risk of dissection [8, 9]. Current guidelines recommend prophylactic aortic root replacement at a threshold diameter of 50 mm. In case of high risk for rupture e.g. due to a growing rate of at least 5 mm per year, elective surgery is recommended at an external diameter of 50 mm [10] or even less (45 mm) in patients with risk factors.

Several biomarkers for progression of aortic dilatation, such as transforming growth factor-β and fibrillin-1 fragments, have been investigated, but none are routinely used in clinical practice [11]. Four-dimensional phase contrast magnetic resonance imaging (4D flow MRI) is an advanced imaging technique, which allows for acquisition of morphological images and velocity data. 4D flow MRI thus enables visualization and quantification of complex blood flow patterns in the thoracic aorta [12, 13]. Recent 4D flow MRI studies showed promising results in Marfan patients supporting the notion that pathological blood flow profiles may contribute to aneurysm formation [14, 15]. Further longitudinal studies are needed to assess the potential of 4D flow MRI as a potential imaging biomarker for progression of aortic dilatation.

However, these imaging biomarkers are not yet established in clinical routine or current guidelines. Therefore, absolute aortic diameter and the aortic growth rate remain the most important predictors for aortic dissection in Marfan patients [16].

As a result, annual cross-sectional imaging is mandatory for monitoring aortic root dimensions in Marfan patients to determine if and when aortic root replacement is indicated [17, 18]. Imaging modalities that allow precise, reproducible, operator-independent and standardized assessment of the aorta are crucial in the follow-up of Marfan patients.

Transthoracic echocardiography (TTE), multidetector computed tomography (MDCT) and magnetic resonance imaging (MRI) are available for non-invasive imaging of the aortic root [10]. However, echocardiography cannot assess the entire aorta and is highly operator-dependent while MDCT uses ionizing radiation and requires application of iodinated contrast [19]. Magnetic resonance angiography (MRA) does not require ionizing radiation or iodinated contrast. Excellent image quality covering the entire aorta can be acquired in an observer-independent fashion [20, 21]. Therefore, MRA is recommended for imaging of the entire thoracic aorta in patients with congenital heart disease.

Unfortunately, there is no consensus, on whether the aortic wall should be included or excluded in the aortic diameter measurements when using MRA for monitoring of aortic root dimensions in patients with genetic aortic disease such as Marfan syndrome. The guidelines for the diagnosis and management of thoracic aortic disease recommend outer-to-outer edge measurements, based on the idea that inner-to-inner edge measurements may not accurately reflect the true diameter in case of mural atherosclerotic plaques, thrombi or aortic wall inflammation [10]. In contrast, the Society for Cardiovascular Magnetic Resonance board of trustee’s task force on standardized image interpretation recommends reporting the inner diameter of the aorta [22]. Consequently, recent studies regarding MRI-derived aortic measurements used different approaches: some authors measured inner-to-inner diameters [20], other authors measured outer-to-outer diameters [21, 23] and some authors failed to detail their measurement approach [24].

Hence, there is a lack of both: consensus among different guidelines as well as systematic studies regarding the issue whether the aortic wall should be included in magnetic resonance angiography (MRA)-derived aortic diameter measurements. Therefore, we aimed to compare the reliability of aortic inner-to-inner and outer-to-outer edge measurements of non-contrast MRA in Marfan patients.

## Material and methods

### Study population

We included forty consecutive adult patients (18 males, 22 females; age range 18–57 years; mean 31.2 ± 12 years; median 27 years) with confirmed Marfan syndrome prior to aortic surgery in this retrospective study. All patients underwent both non-contrast MRA and echocardiography on the same day between January and July 2015. Marfan diagnosis was established according to the latest Ghent nosology as well as genetic analyses with sequencing of the *FBN1* gene [3, 25]. We excluded Marfan patients younger than 18 years or with prior aortic root surgery. The local institutional review board approved our retrospective single-centre study and waived the requirement for informed consent.

### Non-contrast MRA

Non-contrast balanced steady-state free precession (bSSFP) MRA was performed using a 1.5 T system equipped with a five-channel coil for cardiac imaging (Achieva, Philips Medical Systems). ECG leads were placed in a typical manner for cardiac triggering. Scout images were acquired in axial, coronal and sagittal orientation. ECG-gated non-contrast 2D MRA with sensitivity encoding (SENSE) was acquired in the transversal and coronal plane as well as in para-sagittal orientation aligned with the curvature of the aortic arch [26, 27]. Image acquisition was triggered to the end-diastolic phase of the cardiac cycle to minimize motion artefacts during end-expiratory breath-hold. Image parameters of para-sagittal orientation were as follows: TR/TE, 3.5/1.75 ms; flip angle, 90°; field of view, 450 mm (FH) × 390 mm (AP) × 102 mm (RL); acquired matrix, 268 × 234; acquired voxel size: 1.68 mm × 1.66 mm × 10 mm; reconstructed voxel size 1.4 mm × 1.4 mm × 10 mm; slice thickness, 10 mm with a gap of − 5 mm (i.e. 5 mm overlap of slices for gapless coverage); SENSE factor: 2. Number of slices: 20; acquisition time 18–22 s for each stack, depending on the individual heart rate and breath-holding capability.

### MR image evaluation

Anonymized non-contrast MRA images were presented in random order to two radiologists with four and six years of experience in cardiovascular imaging, respectively. All images were interpreted on state-of-the-art RIS/PACS workstations (Centricity™ RIS-i 4.2 Plus, GE General Electric Company).

**Fig. 1:**
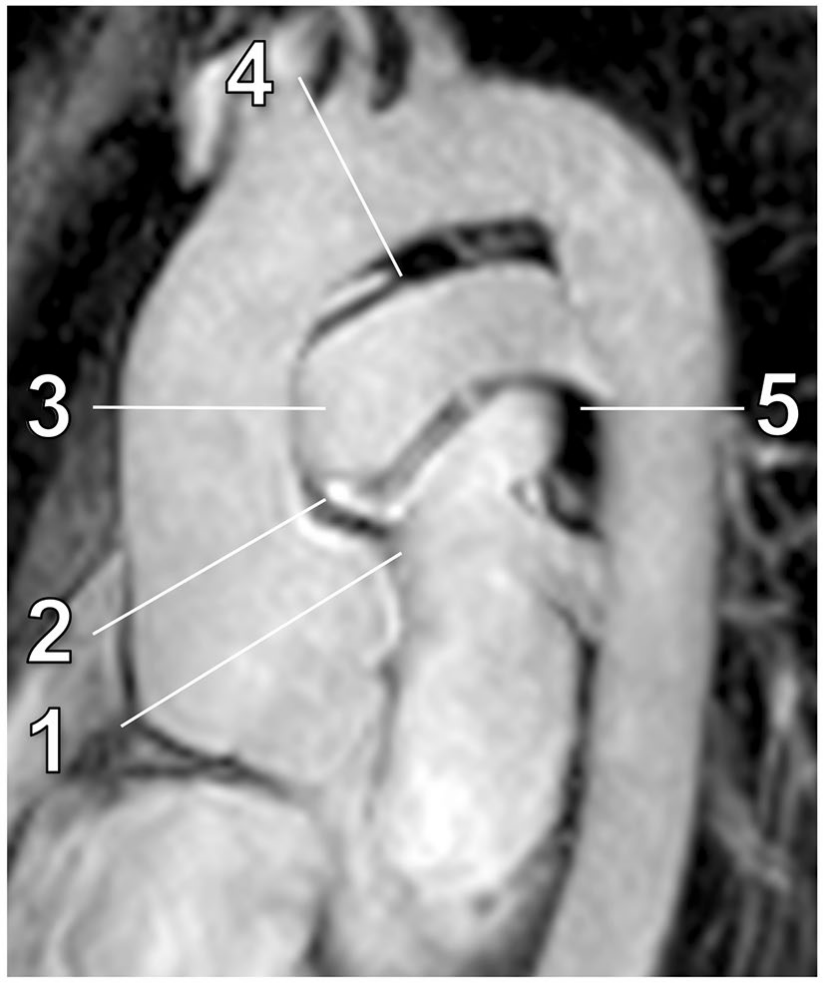
Para-sagittal ECG-gated non-contrast MRA of the thoracic aorta with in a 28-year-old man with confirmed Marfan syndrome. Indicated measurement levels from *proximal* to *distal*: sinuses of Valsalva (1), sinotubular junction (2), ascending aorta (3), aortic arch (4) and descending aorta (5)

Image quality assessments and diameter measurements were performed at the sinuses of Valsalva, sinotubular junction, ascending aorta at the level of the pulmonary trunk, aortic arch between the branching of left carotid and left subclavian artery, and descending aorta at level of the pulmonary trunk as illustrated in Fig. 1 [10].

Image quality of para-sagittal non-contrast MRA images was assessed by both readers individually on a four-point scale regarding sharp anatomic delineation of inner and outer edges of the vessel walls at all levels [21]:

- 1 = excellent definition with sharp delineation and high contrast
- 2 = well defined with good contrast
- 3 = poorly defined with low contrast
- 4 = not visualized

Visibility of a segment was rated to be of diagnostically acceptable image quality if the score was ≤ 2. Thus, readers were confident that diameter measurements could be performed with diagnostic confidence from the visualized segment. Further, both readers analysed the aortic lumen for thrombi to prevent biased image quality evaluation and diameter measurements.

Diameter measurements were performed by both readers individually at all levels on identically orientated para-sagittal non-contrast MRA source images perpendicular to the blood-filled lumen. Readers were free to choose appropriate slices displaying the maximal profile of the aorta from the stacks of para-sagittal images. No secondary multiplanar reformations (MPR) were used for the comparison of the two measurement techniques. Using the identically oriented para-sagittal images avoided user influence introduced by individually performed MPRs and allowed for assessment of only the differences that are attributed to the different measurement techniques [23, 26, 27].

Inner-to-inner and outer-to-outer diameters were measured three times: for assessment of intraobserver agreement, reader 1 performed two measurements, with an interval of four weeks between the first and second measurement. For assessment of interobserver agreement, reader 2 performed a third measurement. Inner-to-inner and outer-to-outer diameter measurements were performed in two different reading sessions with a two-week time lag between assessments of the same case to avoid recall bias.

### Echocardiography

All Marfan patients underwent a 2D transthoracic echocardiographic examination (TEE) on the same day of the non-contrast MRA study as part of their routine screening at our University Heart Centre. Two readers with ≥ 10 years of echocardiographic experience evaluated TTE recordings. End-diastolic aortic root diameters were determined using the leading-edge method in the parasternal long axis view at the level of the sinuses of Valsalva [28]. Echocardiography was performed with a commercially available ultrasound system (EPIQ CVx, Philips, Andover, MA, USA). Aortic diameters at the level of sinuses of Valsalva measured by echocardiography were compared to measurements obtained from bSSFP MRA.

### Statistical analysis

Means and standard deviations of subjective image quality scoring were calculated for inner and outer vessel wall edges images for both readers and for the average of both readers. We do report these results as means ± standard deviations because they are more informative than medians and interquartile ranges. Comparison of the subjective image quality of the anatomic delineation of inner and outer vessel wall edges was performed using the Wilcoxon signed-rank test for paired samples. A weighted k statistic was performed to assess interobserver variability. A k value of 0.81–1.00 indicated excellent agreement; a k value of 0.61- 0.80, substantial agreement; a k value of 0.41–0.60, moderate agreement; a k value of 0.21–0.40, fair agreement; and a k value of 0.00–0.20, slight agreement [29].

Intraclass correlation coefficients (ICC) were calculated to assess intraobserver and interobserver correlation of measurements obtained from inner-to-inner and outer-to-outer measurements. Bland–Altman analysis was used to assess intra- and interobserver agreement between measurements obtained from inner-to-inner and outer-to-outer measurements. A two-sided t test was performed for comparison of mean differences (bias) and F-test for comparison of variances.

Inner-to-inner diameter measurements were compared with outer-to-outer diameter measurements using Bland–Altman analysis. Both inner-to-inner and outer-to-outer diameter measurements were compared with echocardiographic measurements using Bland–Altman analysis. A two-sided paired t test was used to determine significant differences between measurements obtained from 2D bSSFP MRA and echocardiography.

*P-*values < 0.05 were considered as statistically significant. Statistical analysis was performed using MedCalc for Windows, version 12.7.8.0 (MedCalc Software).

## Results

All non-contrast MRA studies were performed with sufficient diagnostic image quality and no rating of 4 (= not visualized) was given. Also, for inner-to-inner measurements only scores of 1 and 2 were given. None of the patients had an aortic dissection. None of the patients had mural thrombi and all studies were included in the evaluation.

### MRA image quality of vessel wall edges

**Fig. 2:**
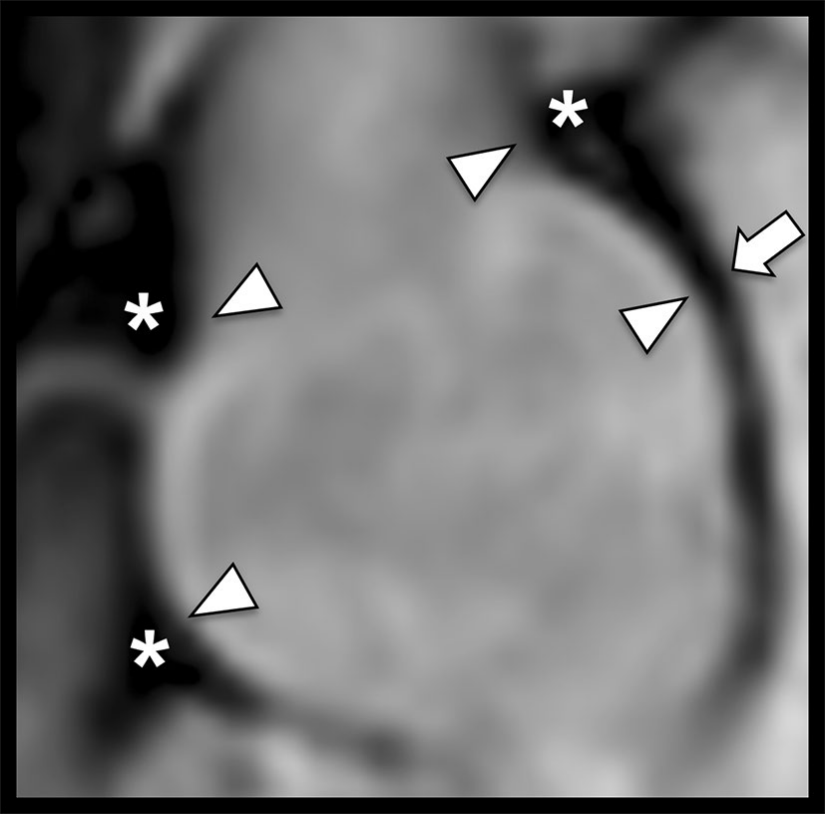
Para-sagittal non-contrast MRA of the aortic root in a 29-year-old man with Marfan syndrome illustrating the advantage of inner diameter measurements. Note the clear delineation of the inner vessel contour at the level of the sinotubular junction and sinuses of Valsalva (arrowheads) due to stark contrast of the hypointense vessel wall to the hyperintense vessel lumen. Compare the hypointense outer contour of the aorta that is obscured by hypointense perivascular tissue (asterisks). A clear delineation of the outer vessel wall is only possible at the level of the sinuses of Valsalva if adjacent to the hyperintense lumen of the left atrium (arrow). Both readers rated the inner vessel wall delineation for the sinuses of Valsalva and sinotubular junction as excellent (grade 1) whereas the outer vessel wall of the sinuses of Valsalva was rated as well defined (grade 2) and sinotubular junction as poorly defined with low contrast (grade 3). Both observers measured identical inner diameters (40 mm vs. 40 mm; bias 0 mm) and deviating outer diameters (48 mm vs. 44 mm; bias 4 mm) for the sinuses of Valsalva

Non-contrast MRA image quality ratings of the anatomical delineation of the vessel wall edges were significantly higher for inner vessel wall edges compared to outer vessel wall edges at all levels of measurement (all p < 0.001) (Fig. 2 and Table 1).

**Table 1:** Qualitative image quality scores for delineation of outer vs. inner vessel walls of the thoracic aorta

Aortic level	Average	Reader 1	Reader 2	Weighted κ (95% CI)
Sinuses of Valsalva				
Inner	1.5 ± 0.5	1.5 ± 0.6	1.5 ± 0.6	0.86 (0.70–1.0)
Outer	2.1 ± 0.7	2.1 ± 0.7	2.1 ± 0.8	0.78 (0.62–0.93)
P-value (t-test)	** < 0.001**	** < 0.001**	** < 0.001**	NA
Sinotubular junction				
Inner	1.6 ± 0.5	1.6 ± 0.5	1.5 ± 0.5	0.74 (0.53–0.95)
Outer	2.3 ± 0.6	2.3 ± 0.7	2.3 ± 0.6	0.62 (0.42–0.82)
P-value (t-test)	** < 0.001**	** < 0.001**	** < 0.001**	NA
Ascending aorta				
Inner	1.3 ± 0.5	1.3 ± 0.6	1.3 ± 0.6	0.82 (0.65–0.99)
Outer	2.1 ± 0.6	2.1 ± 0.6	2.2 ± 0.6	0.79 (0.57–1.0)
P-value (t-test)	** < 0.001**	** < 0.001**	** < 0.001**	NA
Mid-aortic arch				
Inner	1.2 ± 0.4	1.3 ± 0.5	1.2 ± 0.4	0.86 (0.69–1.0)
Outer	2.1 ± 0.6	2.1 ± 0.6	2.1 ± 0.6	0.79 (0.60–0.97)
P-value (t-test)	** < 0.001**	** < 0.001**	** < 0.001**	NA
Descending aorta				
Inner	1.2 ± 0.4	1.2 ± 0.5	1.1 ± 0.3	0.85 (0.65–1.0)
Outer	2.4 ± 0.5	2.4 ± 0.5	2.4 ± 0.5	0.84 (0.66–1.0)
P-value (t-test)	** < 0.001**	** < 0.001**	** < 0.001**	NA

T-test was performed for comparison of mean differences. Significant differences are in bold (significant at *p* < 0.05)

A four point-scale regarding sharp anatomic delineation of inner and outer edges of the vessel was used: 1 = excellent definition with sharp delineation and high contrast; 2 = well defined with good contrast; 3 = poorly defined with low contrast; 4 = not visualized

*NA*  not  applicable

### Intraobserver agreement of MRA-derived measurements

Intraobserver correlation was good for both methods at all measurement levels (all r > 0.8) (Table 2).

**Table 2:** Intraobserver variance of inner vs. outer aortic measurements as described by Bland and Altman

	Sinuses of Valsalva	Sinotubular junction	Ascending aorta	Aortic arch	Descending Aorta
Outer-outer edge					
Mean difference (mm)	0.80	0.40	0.13	0.65	0.40
Limits of agreement (mm)	− 4.2 to 5.8	− 4.0 to 4.8	− 3.5 to 3.7	− 3.1 to 4.3	− 3.6 to 4.4
SD (mm)	2.5	2.2	1.9	2	2.1
Variance (mm^2^)	6.4	5.0	3.4	3.9	4.2
ICC (r)	0.91	0.89	0.95	0.83	0.83
Inner-inner edge					
Mean difference (mm)	0.43	0.10	0.52	0.35	− 0.15
Limits of agreement (mm)	− 3.1 to 3.9	− 3.0 to 3.2	− 2 to 3.1	− 3.1 to 3.8	− 4.4 to 4.2
SD (mm)	1.8	1.6	1.3	1.7	2.2
Variance (mm^2^)	3.1	2.5	1.7	3.1	4.8
ICC (r)	0.95	0.95	0.97	0.86	0.86
P value (t test)	** < 0.001**	** < 0.001**	** < 0.001**	** < 0.001**	** < 0.001**
P value (F test)	**0.03**	**0.03**	**0.03**	0.46	0.65

Intraclass correlation coefficient (ICC) values are given for outer and inner measurements. T-test was performed for comparison of mean differences and F-test for comparison of variances

Significant differences are in bold (significant at *p* < 0.05)

**Fig. 3:**
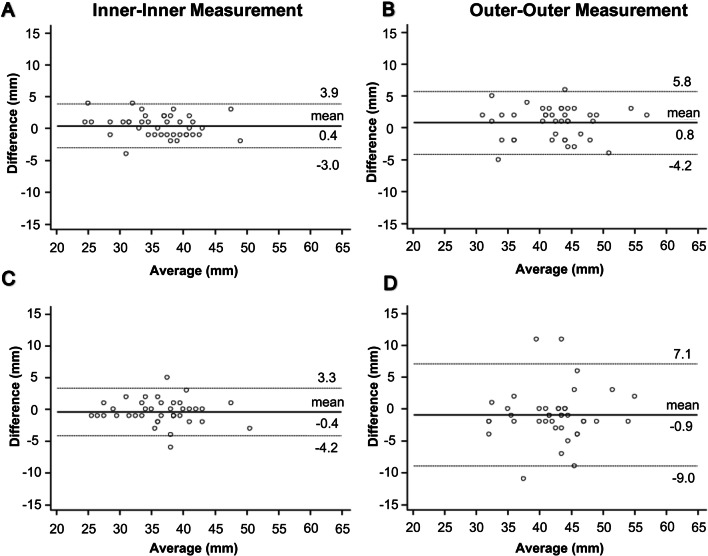
Intra- and interobserver agreement of inner vs. outer aortic diameter measurements using non-contrast MRA at the sinuses of Valsalva. **a**, **b **Bland–Altman plots of intraobserver agreement demonstrate a significantly smaller intraobserver variance of **a** inner measurements compared to **b** outer measurements (p < 0.001). **c**, **d **Analyses of interobserver agreement measurements also demonstrate a significantly smaller interobserver variance of **c **inner measurements compared to **d **outer measurements (p = 0.001). Middle solid line indicates mean bias of diameter measurements. Dotted lines indicate limits of agreement

Bland–Altman analyses revealed a significantly smaller (p < 0.001) intraobserver bias for inner-to-inner measurements at the sinuses of Valsalva (mean difference: 0.4 mm) compared to outer-to-outer measurements (mean difference: 0.8 mm) (Fig. 3a, b). The intraobserver bias was significantly smaller for inner-to-inner measurements compared to outer-to-outer measurements also at all other measurement levels (all p < 0.001) (Table 2).

Bland–Altman analyses display a significantly smaller (p = 0.03) intraobserver variance for inner-to-inner measurements at the sinuses of Valsalva (95% limits of agreement: − 3.1 to 3.9 mm) compared to outer-to-outer measurements (95% limits of agreement: − 4.2 to 5.8 mm) (Fig. 3a, b, Supplemental Fig. 1). The intraobserver variance was significantly smaller for inner-to-inner measurements compared to outer-to-outer measurements also at the sinotubular junction (p = 0.03) and the ascending aorta (p = 0.03). There was no significant difference in intraobserver variances at the level of the aortic arch (p = 0.46) and the descending aorta (p = 0.65) (Table 2).

### Interobserver agreement of MRA-derived measurements

Interobserver correlation was higher for inner-to-inner measurements (all r > 0.8) than for outer-to-outer measurements, except for the aortic arch and descending aorta where interobserver correlation was comparable to outer-to-outer measurements (Table 3).

**Table 3:** Interobserver variance of inner vs. outer aortic measurements as described by Bland and Altman

	Sinuses of Valsalva	Sinotubular junction	Ascending aorta	Aortic arch	Descending aorta
Outer-outer edge					
Mean difference (mm)	− 0.93	0.50	− 0.60	0.40	0.30
Limits of agreement (mm)	− 8.9 to 7.1	− 7.5 to 8.5	− 11.4 to 10.2	− 3.0 to 3.8	− 3.7 to 3.3
SD (mm)	4.1	4.1	5.5	1.8	1.8
Variance (mm^2^)	16.9	16.8	30.1	3.1	3.1
ICC (r)	0.75	0.67	0.48	0.83	0.88
Inner-inner edge					
Mean difference (mm)	− 0.43	0.18	0.15	− 0.05	0.20
Limits of agreement (mm)	− 4.2 to 3.3	− 5.6 to 5.9	− 4.7 to 4.4	− 2.7 to 2.8	− 2.7 to 2.5
SD (mm)	1.9	2.9	2.3	1.4	1.3
Variance (mm^2^)	3.7	8.8	5.4	2.0	1.7
ICC (r)	0.94	0.82	0.89	0.89	0.92
P value (t test)	** < 0.001**	** < 0.001**	** < 0.001**	** < 0.001**	** < 0.001**
P value (F test)	** < 0.001**	** < 0.05**	** < 0.001**	0.16	0.07

Intraclass correlation coefficient (ICC) values are given for outer and inner measurements. T-test was performed for comparison of mean differences and F-test for comparison of variances

Significant differences are in bold (significant at *p* < 0.05)

Bland–Altman analyses revealed a significantly smaller (p < 0.001) interobserver bias for inner-to-inner measurements at the sinuses of Valsalva (mean difference: − 0.4 mm) compared to outer-to-outer measurements (mean difference: − 0.9 mm) (Fig. 3c, d). The interobserver bias was significantly smaller for inner-to-inner measurements compared to outer-to-outer measurements also at all other measurement levels (all p < 0.001) (Table 3).

Bland–Altman analyses further revealed a significantly smaller (p = 0.001) interobserver variance for inner-to-inner measurements at the sinuses of Valsalva (95% limits of agreement: − 4.2 to 3.3 mm) compared to outer-to-outer measurements (95% limits of agreement: − 9 to 7.1 mm) (Fig. 3c, d). The interobserver variance was significantly smaller for inner-to-inner measurements compared to outer-to-outer measurements also at the sinotubular junction (p < 0.05) and the ascending aorta (p < 0.001). There was no significant difference in interobserver variances at the level of the aortic arch (p = 0.16) and the descending aorta (p = 0.07) (Table 3).

### Comparison of MRA-derived inner vs. outer aortic measurements

**Fig. 4:**
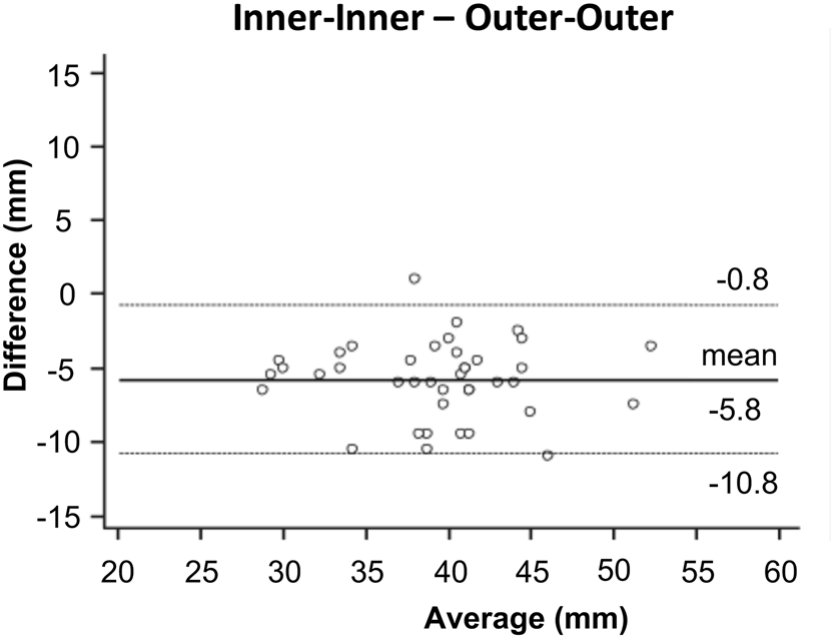
Bland–Altman comparison of the measured diameters at the sinuses of Valsalva assessed by inner and outer non-contrast MRA measurements. The plot illustrates a significant difference of − 5.8 ± 2.6 mm for inner measurements compared to outer measurements (p < 0.0001). Middle solid line indicates mean bias of diameter measurements. Dotted lines indicate limits of agreement

Bland–Altman analyses of the measured diameters at the sinuses of Valsalva revealed a significantly higher diameter of outer measurements compared to inner diameter measurements with a mean of − 5.8 ± 2.6 mm (p < 0.001) Fig. 4). The measured aortic diameter was significantly higher for outer measurements compared to inner measurements also at all other measurement levels (all p < 0.001) (Table 4).

**Table 4:** Comparison of aortic diameters as determined by inner and outer measurements

	Sinuses of Valsalva	Sinotubular junction	Ascending aorta	Aortic arch	Descending aorta
Inner-inner (mm)	36.1 ± 5.4	27.4 ± 4.7	25.8 ± 4.7	20.7 ± 2.9	20.5 ± 3.1
Outer-outer (mm)	42.3 ± 5.5	32.2 ± 4.6	30.6 ± 4.6	25.1 ± 3.0	25.3 ± 3.4
Mean difference (mm)	− 6.2 ± 2.4	− 4.8 ± 1.6	− 4.9 ± 2.6	− 4.4 ± 1.9	− 4.8 ± 1.5
P-value (t-test)	** < 0.001**	** < 0.001**	** < 0.001**	** < 0.001**	** < 0.001**

T-test was performed for comparison of mean differences

Significant differences are in bold (significant at *p* < 0.05)

The mean difference between both methods ranged from − 4.4 ± 1.9 mm in the aortic arch to − 6.2 ± 2.4 mm at the sinuses of Valsalva (Table 4).

### Comparison of MRA-derived inner and outer measurements vs. echocardiography

**Fig. 5:**
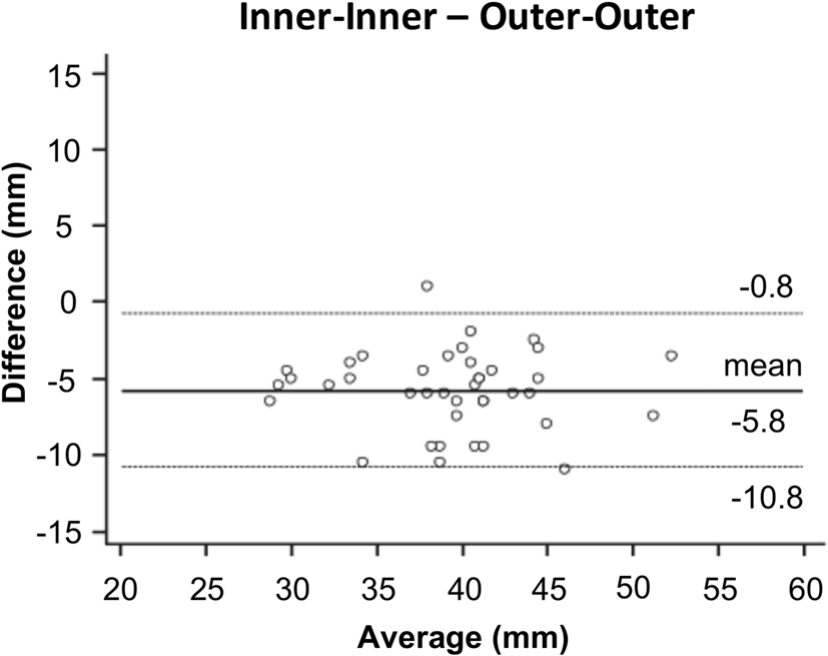
Bland–Altman comparison of inner and outer non-contrast MRA diameter measurements vs. echocardiographic leading-edge measurements at the sinuses of Valsalva. The plots reveal a difference of **a** 1.1 ± 2.3 mm for inner MRA measurements (p = 0.014) and a difference of **b** 6.9 ± 3.1 mm for outer MRA measurements (p < 0.001) when compared to echocardiography. Keep in mind the para-sagittal orientation of MRA-derived measurements vs. parasternal long axis-view orientation of echocardiographic measurements.* Middle solid line* indicates mean bias of diameter measurements. *Dotted lines* indicate limits of agreement

Bland–Altman analyses of the measured diameters at the sinuses of Valsalva revealed a significantly higher diameter of MRA-derived outer-to-outer measurements (35 ± 5 mm) when compared to echocardiographic measurements (42 ± 6 mm), resulting in a mean difference of 6.9 ± 3.1 mm (p < 0.001). MRA-derived inner-to-inner measurements at the sinuses of Valsalva (36 ± 5 mm) were also significantly higher (p = 0.014) when compared to echocardiographic measurements (35 ± 5 mm), but with a smaller mean difference of 1.1 ± 2.3 mm (Fig. 5).

### Impact on clinical decision-making

**Fig. 6:**
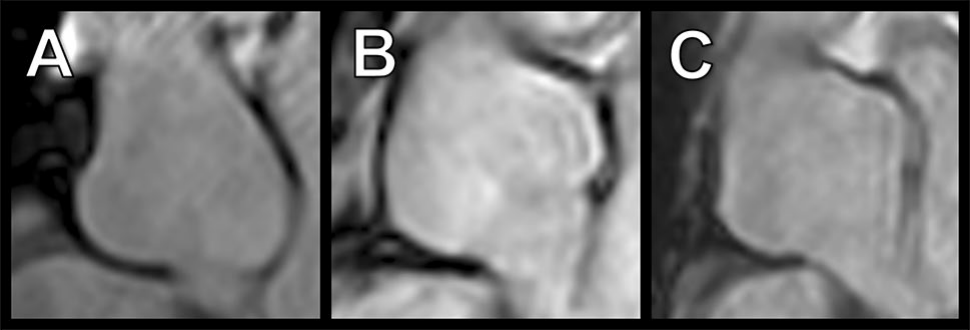
Para-sagittal non-contrast MRA of three Marfan patients with aortic root aneurysms with diameters larger than 45 mm. **a**–**c** All three aneurysms were correctly identified using MRA-derived inner-to-inner measurements. These findings were confirmed by echocardiography and all patients underwent prophylactic aortic root replacement. Both readers rated the inner vessel wall delineation for the sinuses of Valsalva as superior when compared to outer vessel wall delineation. Both observers measured comparable inner diameters: A: 47 vs. 48 mm; B: 51 vs. 51 mm; C: 50 vs. 50 mm which were similar to diameters obtained by echocardiography: A: 46 mm; B: 49 mm; C: 51 mm. Outer diameters showed a higher deviation: A: 50 vs. 52 mm B: 55 vs. 59 mm; C: 53 vs. 57 mm

Both readers correctly identified aortic root aneurysms ≥ 45 mm in three of the 40 included patients (8%) with additional risk factors using MRA-derived inner-to-inner measurements (Fig. 6). These findings were correlated with echocardiography and all three patients underwent prophylactic aortic root replacement to evade the risk of dissection. Using MRA, both readers measured comparable inner diameters in all three patients for the sinuses of Valsalva (differences: 0, 0, and 1 mm, respectively) and deviating outer diameters (differences: 2, 4, and 4 mm, respectively). Compared with diameters obtained by echocardiography there was a bigger difference for outer diameters than inner diameters (4 and 6 vs. 1 and 2 mm; 6 and 10 vs. 2 and 2 mm; 2 and 6 vs. 1 and 1 mm).

## Discussion

We successfully demonstrated that inner-to-inner measurements are more reliable than outer-to-outer edge measurements when using ECG-gated non-contrast MRA for monitoring of aortic diameters in Marfan patients.

Our results revealed that inner aortic vessel wall edges show a clear demarcation due to the high contrast between the hypointense vessel wall and the hyperintense vessel lumen. In contrast, outer vessel wall edges show a significantly worse demarcation, which is explained by the hypointense vessel wall that is surrounded by also hypointense perivascular tissue, resulting in a more difficult discrimination. The better delineation of the inner vessel wall edges translated in significantly smaller intra- and interobserver biases of the entire thoracic aorta and significantly smaller variances of measurements for the aortic root.

There were no significant differences of variances between inner and outer measurements at the level of the aortic arch and the descending aorta, which might be explained by a clear demarcation of the aortic arch and the straight course of the descending aorta. This observation is in accordance with a recent study demonstrating higher correlation of measurements at the level of the aortic arch and descending aorta compared to other aortic levels [30].

As expected, inner-to-inner diameter measurements resulted in significantly smaller diameters when compared to outer-to-outer measurements. At the sinuses of Valsalva this diameter difference amounted to a statistically significant difference of ~ 6 mm. A diameter difference of 6 mm is clinically relevant, particularly in Marfan patients approaching the threshold diameter for aortic root surgery, thereby impacting on indication of elective aortic root surgery.

Both inner and outer para-sagittal MRA-derived measurements of the sinuses of Valsalva were significantly higher when compared to parasternal long axis echocardiographic leading-edge measurements. Expectedly, the difference between echocardiographic leading-edge diameter and MRA-derived outer diameters (~ 7 mm) was higher and clinically more relevant than the difference from MRA-derived inner diameters (~ 1 mm). The remaining small difference between MRA-derived inner-to-inner diameters and echocardiographic leading-edge diameters is explained by the different angle of incidence and imaging modalities [31]. Taken together, MRA-derived inner-to-inner measurements show a smaller variance and higher agreement with echocardiographic measurements than outer-to-outer measurements. These results regarding aortic diameter assessment are clinically relevant as they are critical for surgical decisions in Marfan patients.

Our study emphasizes the importance of unified standardization regarding the inclusion of the aortic wall in aortic diameter measurements for non-contrast MRA in Marfan patients. According to our results, we propose a standardized report of the internal aortic diameter for non-contrast MRA in Marfan patients. Our findings support the recommendation of the Society for Cardiovascular Magnetic Resonance board of trustee’s task force on standardized image interpretation in cardiovascular magnetic resonance. Of note, the guidelines for the diagnosis and management of thoracic aortic disease recommend the opposite approach. However, both guidelines state that the external diameter is useful to avoid confounding by mural thrombi [10, 22], as it is commonly found in the abdominal but not in the ascending aorta [10, 28]. This is in accordance with our study: none of the included patients had mural thrombi. Therefore, we do advocate internal measurements of the thoracic aorta in Marfan patients when using MRA.

### Study limitations

There are certain limitations to this study. First, measurements were only performed in para-sagittal planes of source images (aligned with the curvature of the aortic arch along the flow axis of the aorta) without using secondary multiplanar reformations. However, using identically orientated source images for both inner-to-inner and outer-to-outer measurements minimizes possible bias introduced by suboptimal secondary reformations and subsequent measurement errors. Assessment of identical source images allows for true assessment of the influence of inner edge vs. outer edge measurement technique on measurement agreement. We acknowledge that secondary reformations such as double oblique techniques may result in different absolute diameters [32]. However, such secondary reformations also introduce operator-dependent biases. Using the identically oriented para-sagittal images avoided user influence introduced by individually performed MPRs and allowed for assessment of only the differences that are attributed to the different imaging and triggering techniques [23, 26, 27]. In every day clinical practice, we use and recommend secondary reformations and/or dedicated cardiac cine MRI in Marfan patients reaching critical aortic root diameters. Secondly, we did not assess other MRA techniques such as other non-contrast 3D-MRA sequences or contrast-enhanced MRA. Another limitation is that we only used bright-blood MR sequences. Dark-blood MR sequences allow for better delineation of the aortic wall and may be better suited for measurements of outer aortic diameter measurements [33, 34]. Future studies addressing the comparison of these MR sequences are warranted.

## Conclusions

In conclusion, inner-to-inner vessel wall measurements in non-contrast MRA provide more reliable diameters when compared to outer-to-outer vessel wall measurements of the aortic root. Therefore, we propose to rely on inner rather than outer aortic wall measurements in non-contrast MRA when monitoring aortic diameters in patients with Marfan syndrome or other thoracic aortic disease.

## Electronic supplementary material

Below is the link to the electronic supplementary material.

Supplemental Figure 1: Intraobserver agreement of inner and outer aortic diameters at the sinuses of Valsalva assessed by observer 2. Bland–Altman plots of intraobserver agreement demonstrate a smaller intraobserver variance of **a** inner measurements compared to **b** outer measurements. Middle solid line indicates mean bias of diameter measurements. Dotted lines indicate limits of agreement (TIF 7997 kb)
