# Supplementary figures and images for: Reliability of non-contrast magnetic resonance angiography-derived aortic diameters in Marfan patients: comparison of inner vs. outer vessel wall measurements
Source: Int J Cardiovasc Imaging. 2020 Apr 20;36(8):1533–42. doi: 10.1007/s10554-020-01850-4 (PMC7381445; doi:10.1007/s10554-020-01850-4)

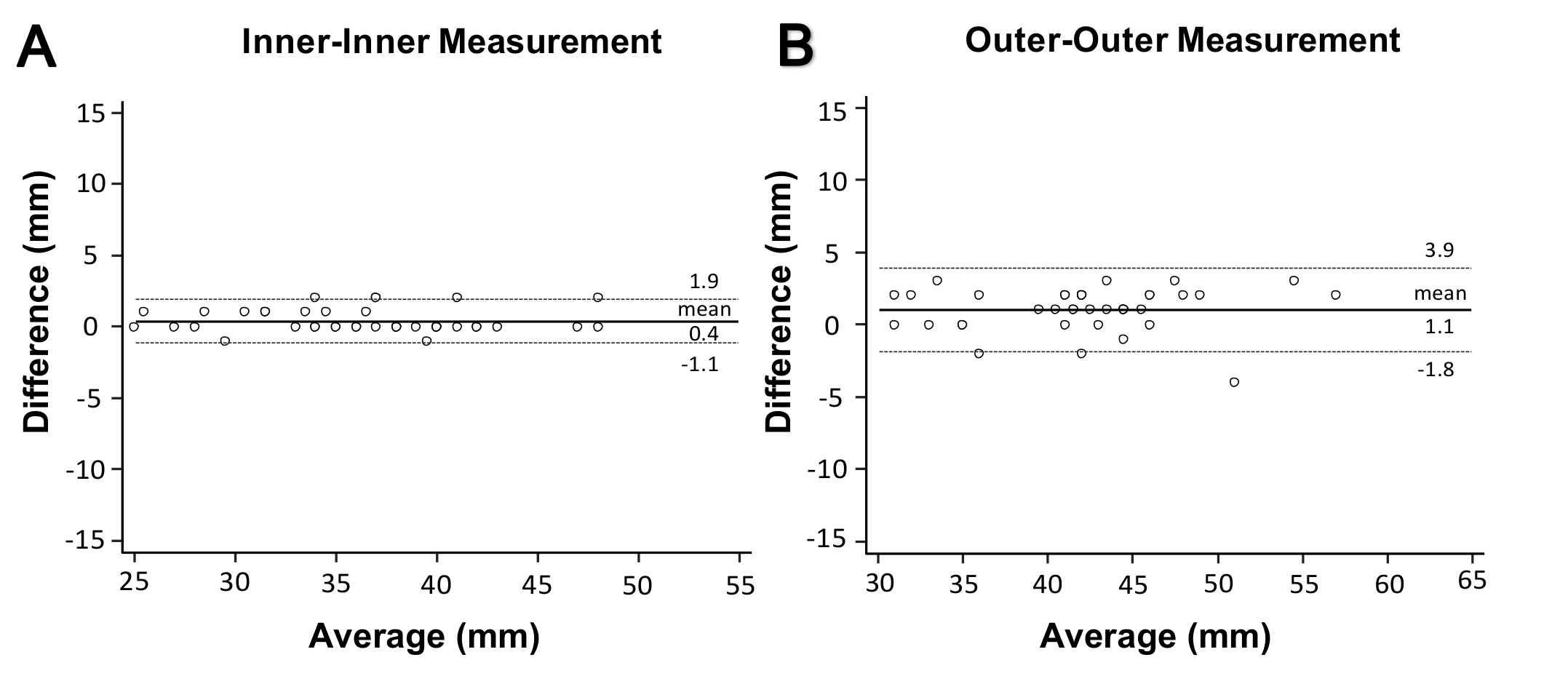

Supplement: Supplementary file 1 — Supplemental Figure 1: Intraobserver agreement of inner and outer aortic diameters at the sinuses of Valsalva assessed by observer 2. Bland–Altman plots of intraobserver agreement demonstrate a smaller intraobserver variance of a inner measurements compared to b outer measurements. Middle solid line indicates mean bias of diameter measurements. Dotted lines indicate limits of agreement (TIF 7997 kb) [file 10554_2020_1850_MOESM1_ESM.tif]
